# Synthesis and kinetic analysis of poly(N-acryloylmorpholine) brushes via surface initiated RAFT polymerization

**DOI:** 10.55730/1300-0527.3528

**Published:** 2022-11-29

**Authors:** Esma MUTLUTÜRK, Tuncer ÇAYKARA

**Affiliations:** 1 Department of Chemistry, Polatlı Faculty of Science and Letters, Ankara Hacı Bayram Veli University, Ankara, Turkey; 2 Department of Chemistry, Faculty of Science, Gazi University, Ankara, Turkey

**Keywords:** Polymer brushes, poly(N-acryloyl morpholine), surface-initiated RAFT polymerization

## Abstract

Polymer brushes are promising many applications as smart materials and biocompatible surfaces. Surface-initiated reversible addition-fragmentation chain transfer (RAFT) polymerization is one of the most effective techniques for synthesis of well-defined polymer brushes. Herein, a biocompatible, uniform and stable poly(N-acryloylmorpholine)-silicon hybrid system was achieved using surface-initiated RAFT polymerization. Evidence of a well-controlled surface-initiated RAFT polymerization was confirmed by a linear increase of number average molecular weight (M_n_) with overall monomer conversions. Water contact angle, ellipsometry, X-ray photoelectron spectroscopy and atomic force microscopy verified the presence of poly(N-acryloylmorpholine) (poly(NAM)) on silicon wafers. The grafting density (σ) and the average distance between grafting points (D) were estimated to be 0.58 chains/nm^2^ and 1.5 nm, respectively. The ratio of D value to radius of gyration (Rg) value is smaller than 1 (D/2Rg < 1), which corresponds to the brush regime of all grafted poly(NAM) films.

## 1. Introduction

Polymer chains attached by one end to a solid interface are called “polymer brushes”. Strong covalent bonding of polymer chains to solid surfaces provide superior characteristics compared to polymer chains in solution. Polymer chains can be decorated on surfaces as low densely grafted “mushroom” or high densely grafted “brush” regime. Polymer brushes have remarkable properties due to their different conformations, thus showing promising applications in tissue engineering, controlled drug delivery, and designing smart materials [1–3].

There are mainly three methods for fabrication of polymer brushes, namely “grafting from”, “grafting to”, and “grafting through” methods. Among these approaches, “grafting from” is usually favored to obtain higher grafting densities and film thickness. In this method, polymerization is initiated on substrate which is modified with a functional group; therefore, chains are grown-up through surface-initiated polymerization [4]. Surface-initiated controlled radical polymerization (SI-CRP) has provided precise control over polymer molecular weight, composition or structure. Nitroxide-mediated polymerization (NMP), atom transfer radical polymerization (ATRP), reversible addition-fragmentation chain-transfer (RAFT) polymerization methods are known as SI-CRP methods for preparation of polymer brushes [5]. Among SI-CRP methods, RAFT polymerization is arguably the most versatile technique. The RAFT method can be performed under mild conditions with various monomers and does not require metal catalysts [6–8]. Different chain control agents (CTA) can be used for polymerization control and they remain active at the end of polymer chains as functional group [9]. High end-group diversity offers to construction macromolecules with different compositions and architectures [10]. RAFT polymerization can be achieved by adding desired chain control agents to conventional free radical polymerization solutions [11]. Because of the difficulty of covalent attachment of control agents to substrates, the polymeric structures prepared on the surface are quite inadequate compared to those synthesized in solution [12]. Silicon wafers are mostly used as substrates for fabrication of polymer brushes. Chemical modification of silicon surfaces for surface-initiated polymerization is the main approach for preparation of polymer brushes. Functionalization of silicon layer with silane-based molecules is usually utilized to form self-assembled monolayers (SAMs) by forming Si-O-Si bonds. However, Si-O-Si bonds that link the monolayer to the surface are partially unstable and susceptible to hydrolysis. In recent years, hydrogen-terminated silicon surfaces are mainly used for fabrication of silicon-based materials. Yet, the hydrogen-terminated surfaces can easily be oxidized by water or oxygen. For this reason, self-assembly monolayers on hydrogen-terminated surfaces can form stable Si-C bonds and prevent oxidation. Alkene molecules can be used to modify hydrogen-terminated silicon surface (Si-H) under heat or UV light. Stability investigation of these monolayers showed that Si-C bond is more stable in organic solvents, acidic and basic conditions, and high temperatures [13–15].

N-acryloylmorpholine is an acrylamide derivative and soluble in polar or low polar solvents [16]. Poly(NAM) is widely used in biological applications due to its biocompatibility [17,18]. Low toxicity of poly(NAM) would be worth exploring further as an in vivo delivery system. In literature, Torchilin and coworkers synthesized liposomes modified with poly(N-acryloylmorpholine) using a dialysis method [19–22]. In another study, photolithographically patterned thermoresponsive poly(N-isopropylacrylamide)-b-poly(N-acryloylmorpholine) brushes were developed on surface using RAFT-mediated copolymerization [23]. Up to now, the free radical polymerization [24] and LRP techniques [25] were used for the synthesis of poly(NAM) in the literature. In this study, more stable Si-C bonding poly(NAM) brushes were fabricated on BPAT-grafted wafers using surface initiated RAFT polymerization and detailed kinetic analysis were examined. Undoubtedly, structurally well-controlled and superior stability compared to conventional Si-O-Si bond and biocompatible polymer brushes will be valuable for new applications.

## 2. Experimental

### 2.1. Materials

Deionized water (> 18 MΩ cm resistivity) was obtained from a Human Power I Scholar-UV water purification system (Seoul, Korea). Silicon wafers (0.5 mm thickness and 125 mm diameter) were obtained from Shin-Etsu, (Handoutai, Japan). All of the other chemicals were purchased from Sigma-Aldrich ( St. Louis, MO, USA ).

### 2.2. Preparation of 1, 2–epoxy–9–decene-ended silicon surfaces

**Figure 1:**
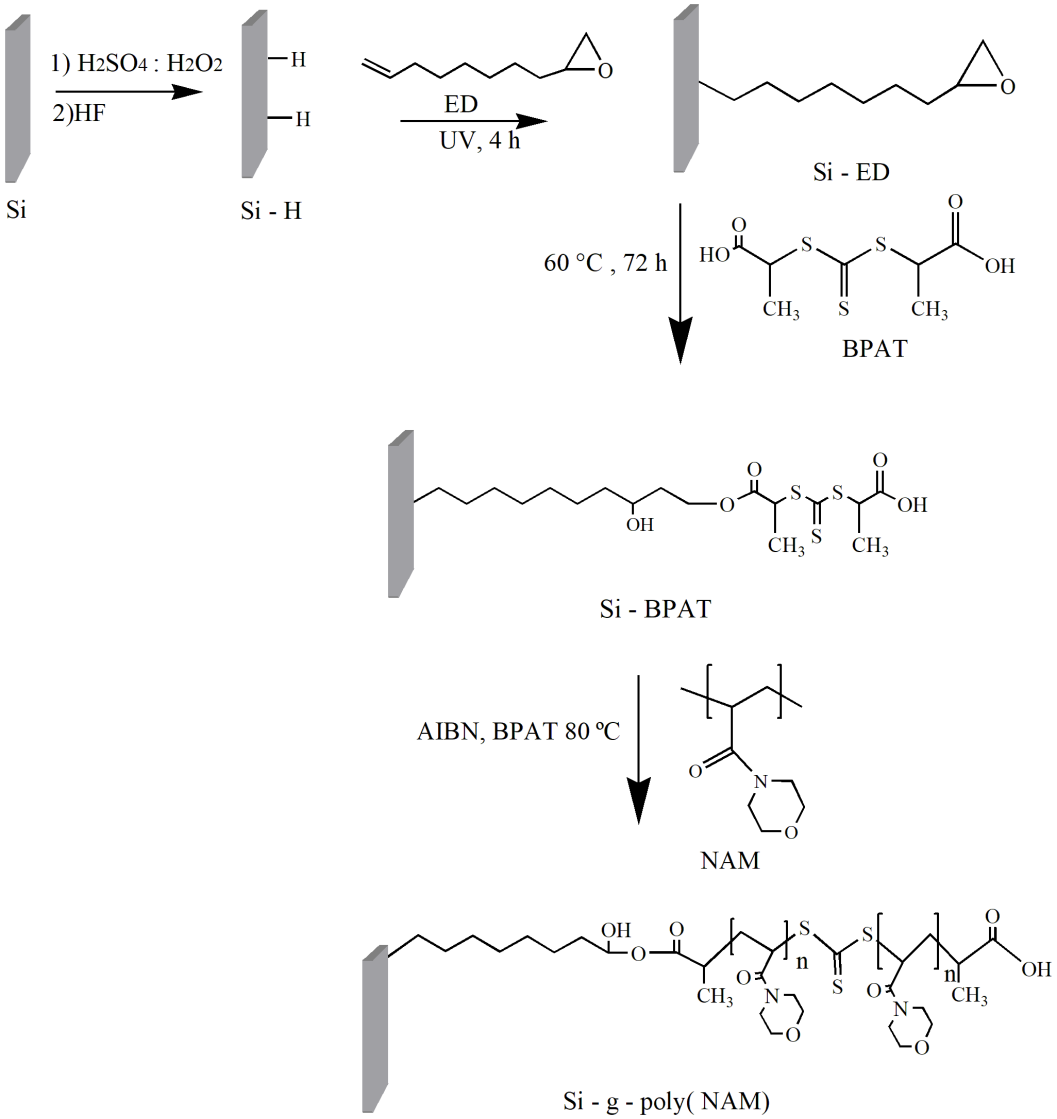
Diagram for synthesis of Si-g-poly(NAM) brushes.

Silicon (111) wafers were cleaned with piranha solution (Piranha solution is mixture of 6:2 (v/v) (H_2_SO_4_: H_2_O_2_)) in 90 º C, for 15 min to remove impurities. Then, surfaces were rinsed with deionized water several times and introduced to a HF solution under nitrogen atmosphere for 10 min [26]. The hydrogen-terminated silicon surfaces (Si-H) were dried with nitrogen, 40 μL ED was added and then sandwiched between two quartz plates in the glove box. The surface was exposed to UV light for 4 h to form stable Si-C bonds on Si-H surfaces (Figure 1). ED functionalized surfaces (Si-ED) were rinsed with cyclohexane and dichloromethane, dried and kept in a nitrogen atmosphere [27].

### 2.3. Synthesis of S, S′-bis(2-propionic acid) trithiocarbonate

For the synthesis of RAFT agent S,S′-bis(2-propionic acid) trithiocarbonate, CS_2_ (0.059 mol) was added to KOH solution (0.082 mol, in 90 mL distilled water ) and stirred for 5 min. 2-bromo propionic acid (0.031 mol) was added dropwise, stirred for 72 h and extracted with dichloromethane (2 × 30 mL), then concentrated with HCl. The yellow solid was extracted with ethyl acetate and dried over anhydrous magnesium sulphate. The solution was filtered and ethyl acetate and solvent were evaporated. The yellow solid was crystallized with toluene, and dried in the oven (supporting information Figures S1 and S2) [28]. ^1^H NMR (300 MHz, CDCI_3_): δ (ppm) 1.6 (d, 3H, -CH3); 4.9 (m, 1H, -CH).

^13^C NMR (300 MHz, CDCI_3_): δ (ppm) 15.9 (-CH3); 48.6 (-CHS); 170.9 (-C=O); 207.0 (-C=S).

(FTIR) ν (cm^−1^): approximately 3200–2360 (s, -O-H), approximately 2970 (s, aliphatic –C-H), 1693 (s, -C=O), 1100 (s, -C=S).

LC-MS: m/z: [M + H]^+^ calcd for C_7_H_10_O_4_S_3_ 254.29; found 254.98 m/z.

### 2.4. Covalent binding of BPAT on 1, 2–epoxy–9–decene-ended surfaces

Si-ED surfaces and BPAT (0.0039 mol) were placed in a nitrogen purged two-neck flask system. The fresh distilled ethyl acetate was added to the mixture and reaction was carried out at 60 °C for 3 days. The surfaces were removed from the flask, washed with ethyl acetate and acetone in a sonic bath. The BPAT modified surfaces (Si-BPAT) were dried and kept in a nitrogen atmosphere.

### 2.5. Synthesis of poly(NAM) brushes via surface-initiated RAFT polymerization

Before the polymerization, a monomer was passed through an alumina-filled column to remove the inhibitor [29]. Polymerization solution was prepared by mixing NAM/free RAFT agent/AIBN (125/1/0.2 molar ratio) and trioxane (NAM/trioxane: 6/1 molar ratio) in dioxane. BPAT-immobilized surfaces were put in the polymer solution and stirred smoothly at 80 ºC under nitrogen atmosphere (Figure 1). After polymerization, substrates were removed and they were rinsed with dioxane, dichloromethane. Bulk polymer was precipitated with excess hexane, filtered and dissolved in dioxane. Precipitation-dissolution process was repeated two times, and then the polymer was dried in vacuum. The Si-g-poly(NAM) surfaces were washed with dioxane and dichloromethane using sonication and dried with nitrogen.

### 2.6. Polymerization kinetic

RAFT polymerization of N-acryloylmorpholine was carried out at 4 different polymerization times (2, 4, 6, 8 h). X-ray photoelectron spectroscopy (XPS), grazing angle Fourier transform infrared spectroscopy (GA-FTIR), water contact angle and ellipsometry measurements were taken for characterization of Si-g-poly(NAM) surfaces. Bulk polymers were characterized by proton nuclear magnetic resonance (^1^H-NMR).

### 2.7. Instrumental techniques

Chemical structure analysis of silicon wafers were achieved using the grazing angle Fourier transform infrared (GA-FTIR) Thermo Nicolet 6700 spectrometer, 128 collecting sample scan, SAGA grazing angle (80°) and ^1^H-NMR analysis (Bruker Avance III 300 MHz). The contact angle measurements were carried using DSA 100, Kruss and deionized water (5 μL, 18 MΩ cm resistivity) at room temperature. For the analysis of film thickness, ellipsometry (DRE, EL X20C) was used. All of the water contact angles and film thicknesses were measured at three different points of surfaces and averaged. Morphology of the surfaces was determined using the atomic force microscopy (AFM Park Systems XE70 SPM), noncontact mode. The X-ray photoelectron spectroscopy (XPS) measurements were performed by an XPS instrument (SPECS XPS) equipped with a Mg Kα X-ray source. After the fitting of the C1s spectra, all the spectra were calibrated in reference to the aliphatic C1s component at a binding energy of 285.0 eV.

## 3. Result and discussion

Synthesis of Si-g-poly(NAM) brushes with Si-C bonds consist of four main steps (Figure 1): i) cleaning of Si (111) surface and HF treatment; ii) UV irradiation of ED sandwiched Si-H surfaces; iii) covalent attachment of BPAT to Si-ED substrates with ring opening reaction; iv) surface-initiated RAFT polymerization of N-acryloylmorpholine on Si-BPAT surfaces.

**Figure 2:**
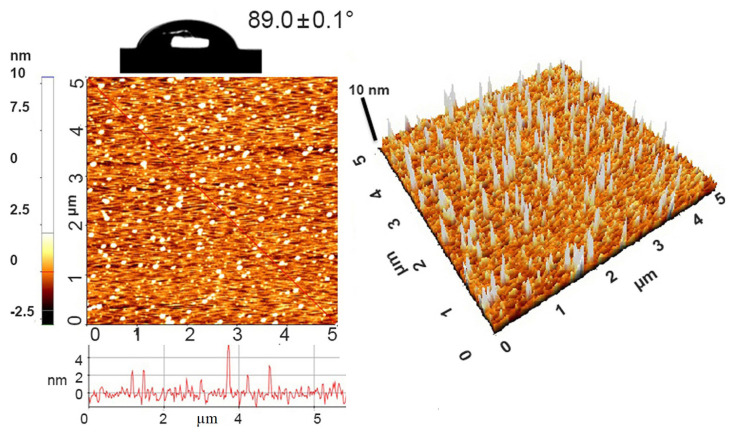
2D-3D AFM images (5 × 5 μm) and water contact angle photograph for Si-H surfaces.

For morphological structure analysis of Si-H surfaces 2D and 3D atomic force microscopy (AFM) images were taken and rough mean square (RMS) value found as 0.4 nm; water contact angle 89.0º (Figure 2).

**Figure 3:**
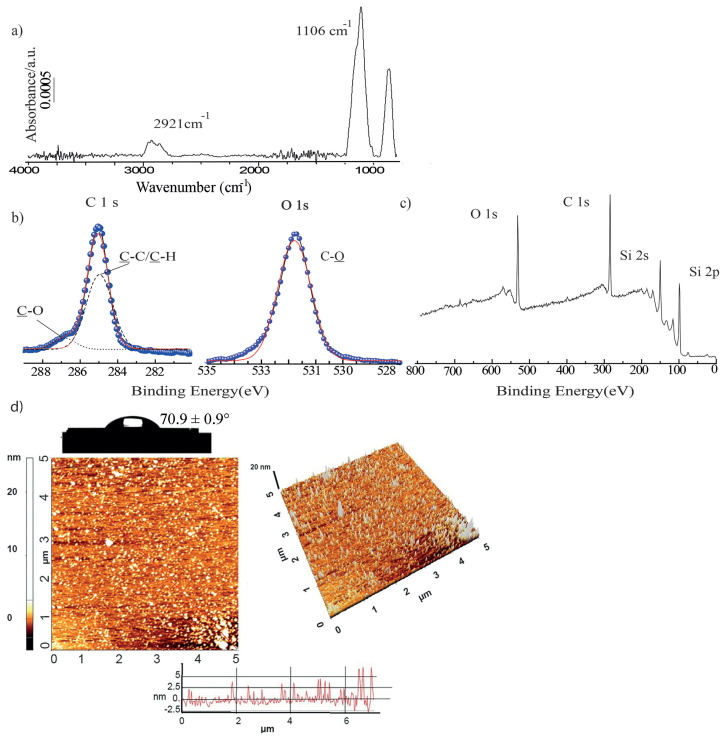
(a) GA-FTIR, (b) O1s, C1, Si2s and Si2p core-level XPS spectra (c) survey scan XPS spectra, (d) 2D-3D AFM images (5 × 5 μm) and water contact angle photograph of Si-ED surfaces.

Optimum irradiation time for covalent bonding of ED molecules, film thickness and water contact angle measurements were found as 4 h (supporting information Figure S4a). In the GA-FTIR spectrum of Si-ED surfaces, the peaks at 2917 cm^−1^ (C-H) and 1106 cm^−1^ (C-O) proved the presence of ED molecules (Figure 3a). Figure 3c shows O 1s, C 1s and Si 2p characteristic peaks in survey scan XPS spectrum. In core-level XPS spectrum, Figure 3c, presence of 532.5 eV (C-O) peaks for O 1s; 286.5 eV (C-O); 285 eV (C-C/ C-H) peaks for C1s indicate the ED binding. Moreover, due to the increased surface roughness, RMS values of surfaces increased from 0.4 nm to 0.9 nm. Water contact angle for Si-ED surface is found as 70.9 ± 0.9° (Figure 3d).

**Figure 4:**
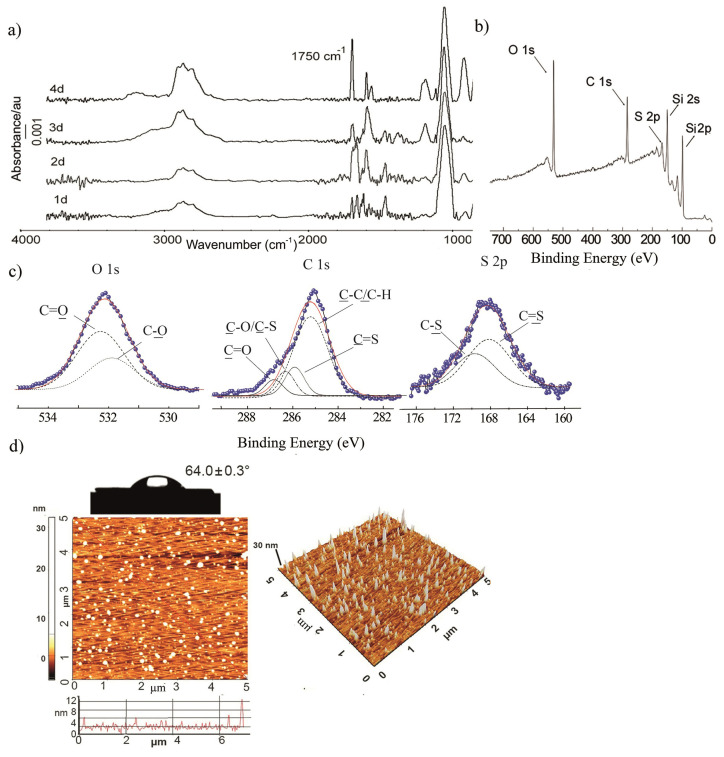
(a) GA-FTIR, (b) survey scan XPS spectra, (c) O1s, C1, S2p, Si2s and Si2p core-level XPS spectra, (d) 2D-3D AFM images (5 × 5 μm) and water contact angle photograph of Si-BPAT surfaces.

In the next step, Si-BPAT surfaces were prepared with different reaction times (24, 48, 72 and 96 h). Since film thickness and water contact angle depends on time, the optimum reaction time for the bonding of the BPAT molecules was determined as 3 days (Figure S4b). BPAT-ended surfaces were characterized using GA-FTIR (Figure 4a), survey scan XPS spectra (Figure 4b), core-level XPS spectra (Figure 4c), AFM and water contact angle measurements (Figure 4d). In GA-FTIR spectrum, the observed specific bands at 3200–2500 cm^−1^ (O-H stretching bands) 1750 cm^−1^ (C=O stretching band) and 1100 cm^−1^ (S=C stretching bands) confirmed the BPAT bonding on the surface. The core-level XPS spectrum of Si-BPAT surface 532.5 eV (C=O) and 532.1 eV (C-O) for O 1s; 287.0 eV (C=O), 286.5 eV (C-O/ C-S); 285 eV (C-C/ C-H) for C 1s and the presence S-C/C=S peaks support efficient binding.

Surface-initiated RAFT polymerization of N-acryloylmorpholine was achieved on Si-BPAT surfaces in presence of AIBN initiator and free BPAT in dioxane at 80 ºC. Poly(NAM) brushes were carried out with different polymerization times. Here, a free RAFT agent was added to the polymerization solution, because polymerization was carried out both in solution and on Si-BPAT surfaces, simultaneously. We assumed that the molecular weight of both free polymers formed on silicon surfaces and in solution is equal to each other [30,31]. Quantity of polymer chains on (1.0 × 1.0 cm) is not enough to determine its molecular weight. Although some polymers can be analyzed by peeling off the surface, it is not easy to apply this process for every polymerization [32]. ^1^H-NMR of poly(NAM) for 6 h polymerization time was given in supporting information, Figure S3.

**Figure 5:**
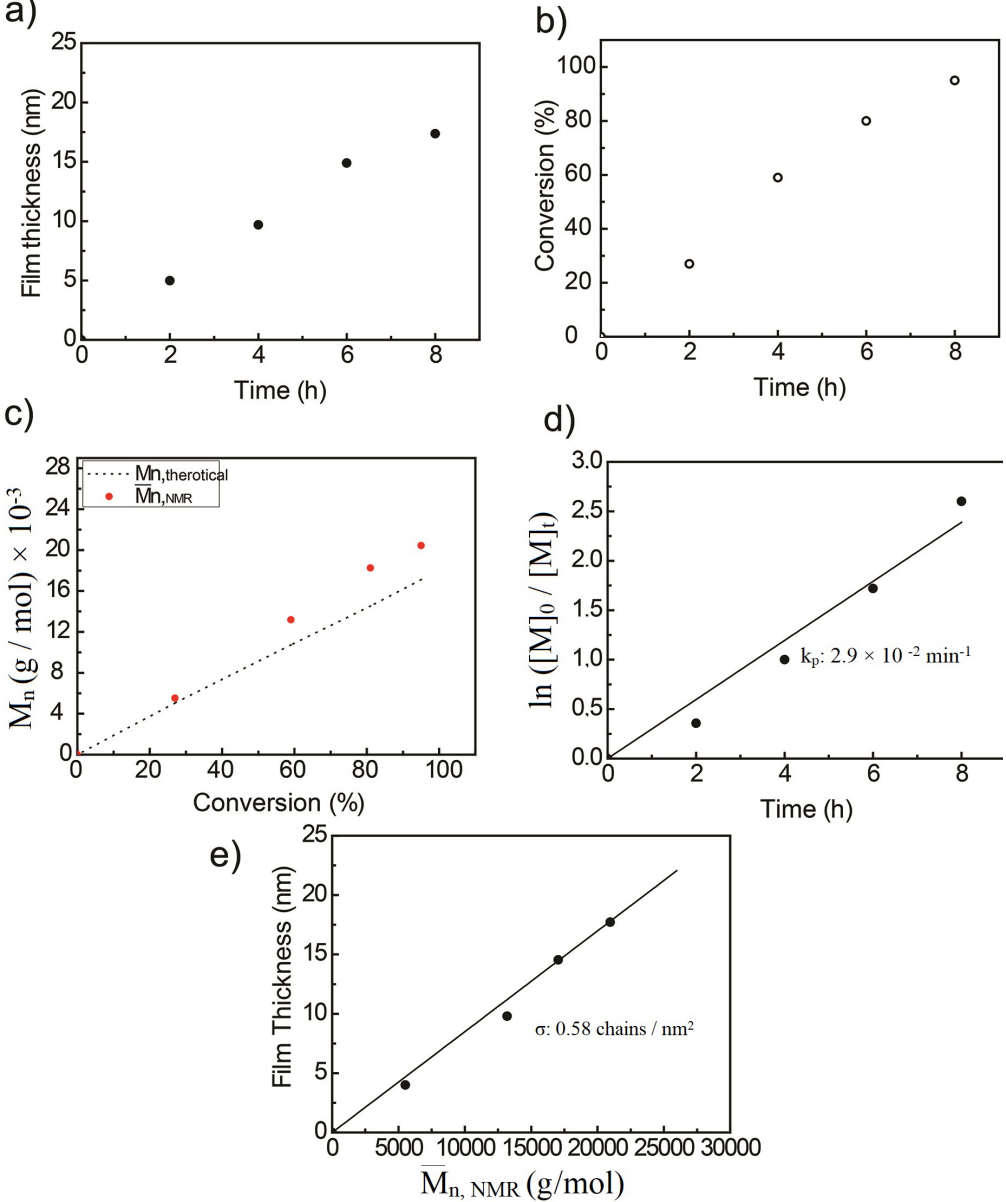
Relationships for (a) film thickness-polymerization time, (b) % conversion-polymerization time, (c) molecular weight – % conversion, (d) ln ([M]_0_/[M]_t_) - polymerization time, (e) film thickness-molecular weights.

For each polymer brush, ellipsometric thicknesses were measured and plotted against the polymerization time. In Figure 5a, the thickness increased linearly within the first 6 hours of polymerization, after that there is deviation from linearity. The linear increase of the surface thickness indicates that the chain growth is controlled. With the increase of polymerization time termination reaction occurs and rate of film thickness slows down [33].

The percent conversion of polymer calculated by ^1^H-NMR plotted against the polymerization time is presented in Figure 5b. The polymerization time increased linearly with the monomer conversion during the first 6 hours which confirms living polymerization behavior. Then, the deviation from the linearity was observed, which indicated free radical reaction.

The theoretical molar mass (M_n, th_) of polymers and monomer conversion calculated using Eqs. 1 and 2, respectively [32,33]. For the absolute molecular mass (M_n, NMR_) calculations, the integral ratio of peaks at 1.1 ppm and 3.7 ppm which corresponds to methyl protons of BPAT and poly(NAM) are used [35]. M_n, th_ and M_n, NMR_ are given in Table 1.


(1) $$Mn,th=\frac{[NAM]o}{\left[CTA\right]}\times M_{NAM}\times \alpha +M_{CTA}$$

where [NAM]_o_ and [CTA] are initial concentrations of monomer the and the RAFT agent, and M_NAM_ and M_CTA_ are molecular weights of the monomer and the RAFT agent.


(2) $$Conversion=1-\frac{{\left(H_{NAM}/H_{TR\text{i}OXANE}\right)}_{t_{t}}}{{\left(H_{NAM}/H_{TR\text{i}OXANE}\right)}_{t_{0}}}$$

The M_n, th_ and M_n, NMR_ were plotted against the polymer conversions (Figure 5c). It is observed that the molecular weights increased linearly with monomer conversion as a result of controlled polymerization [36]. Although linear growth of M_n, th_ conversion can be seen in all experiments, there are some deviations of molecular weights at high monomer conversion due to the fraction of dead chains in high molecular weight [37].

A linearly increasing graph was obtained by plotting ln ([M0]/[M]_t_) versus the polymerization time (Figure 5d). This indicates that the reaction has pseudo first order reaction kinetics [38]. The degree of the reaction is characteristic for the pseudo first order, indicating that the controlled polymerization occurs [39]. From the slope of the graph, the reaction rate constant was found as 2.9 × 10^−2^ h^−1^. A linear relationship is also observed in the film thickness versus molecular weight of the polymer brushes. Slope of the graph was substituted in Eq. 3 and the grafting density was calculated as 0.58 chains/nm^2^ [38]. Here, we accept that the average number of grafting densities for different polymerization times is approximately equal. The number of grafted polymer chains is equal to grafted RAFT agent because monomers were grafted through Si-BPAT surfaces. The same reaction conditions are used to obtain Si-ED and Si-BPAT surfaces for all polymer brush samples. As the polymerization time increased, the number of chains on the surface remained the same, only the chain length/molecular weight ratio increased.


(3) $$\sigma =h\times \rho \times N_{A}/Mn\times 10^{21}$$

σ: grafting density, number of chains/nm^2^; h: film thickness, nm; ρ: polymer density, 1.074 g/cm^3^; N_A_: Avogadro’s number, 6.02 × 10^23^ and Mn: number-average molecular weight.

Using grafting density and Eq. 4, distance between bonding points was calculated as 1.5 nm. Finally, the polymerization degree calculated by ^1^H-NMR was used in Eq. 5 and segment length was found as 3.4 nm [38]. The distance between attachment points is less than twice the radius of the gyration, indicating that the resulting polymers are in the brush structure [40].


(4) $$D={\left(\frac{4}{\pi \sigma }\right)}^{1/2}$$

D: distance between grafting points, nm.


(5) $${\text{R}}_{\text{g}}=\text{b}\;{\left(\frac{{\text{D}\bar{\text{P}}}_{\text{n}}}{6}\right)}^{1/2}$$

Rg: gyration radius, nm; b: chain length, nm; DP_n_: polymerization degree.

**Figure 6:**
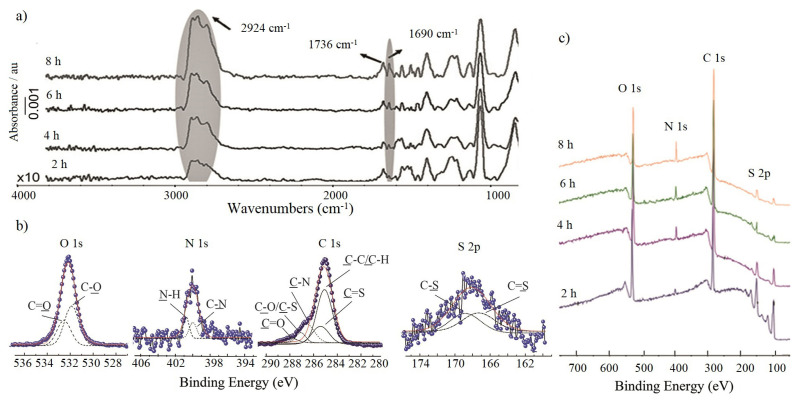
(a) GA-FTIR spectra and (c) core-level XPS spectra of Si-g-poly(NAM) surfaces for all polymerization times, (b) O1s, C1, S2p, Si2s and Si2p of Si-g-poly(NAM) surface.

**Figure 7:**
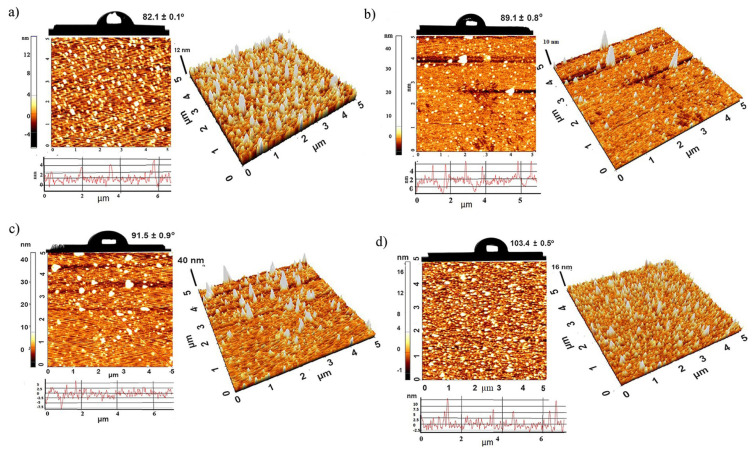
2D-3D AFM images (5 × 5 μm) and water contact angle photograph for polymer brushes synthesized for different reaction time a) 2 h, b) 4 h, c) 6 h, d) 8 h.

For the characterization of polymer brushes GA-FTIR spectrum (Figure 6a), core-level XPS spectrum (Figure 6b), surface scan XPS spectrum (Figure 6c) and AFM images water contact angle measurements (Figure 7) were taken. In GA-FTIR spectrums (bands at 3000–2900 cm^−1^ and 1690 cm^−1^ correspond to aliphatic -C-H and amide carbonyl, respectively. It is obvious that the strength of C-H stretching increases as the polymerization time increases. Figure 6c shows O 1s, C 1s, N 1s and S 2p characteristic peaks in survey scan XPS spectrum for all polymerization times.

XPS surface scan spectrum was used for the determination of elementary composition and the binding energies, given in Table 2. Here, the presence of S2p peaks at 168.2 eV and 167.0 eV suggests that the polymer end group has a CPAD molecule and living polymerization was achieved [38].

The surface morphology of poly(NAM) brushes was analyzed using AFM (Figure 7). RMS roughness is 1.2 ± 0.2 nm and 2.2 ± 0.3 nm for polymer brush synthesized for 2 h and 8 h. RMS values are small enough indicating that homogeneous and uniform surfaces are formed.

Wettability analysis was conducted using water contact angle measurements at three different points for all polymer surfaces (Figure 7). It is observed that the contact angle values increased from 82.1 ± 0.1º to 103.4 ± 0.5º with increasing of the polymerization time.

## 4. Conclusion

In conclusion, a biocompatible, stable and uniform poly(N-acryloylmorpholine)-silicon hybrid system was achieved using surface-initiated RAFT polymerization on ED-ended silicon wafers with the presence of free BPAT. Well-controlled surface-initiated RAFT polymerization was confirmed by a linear increase of number-average molecular weight. The grafting density (σ) and the average distance between grafting points (*D*) were estimated to be 0.58 chains/nm^2^ and 1.5 nm, respectively. Since *D* to the radius of gyration (*R*g) of the corresponding free polymer chains does not exceed 1, films were in the brush regime. Well-defined and biocompatible poly(N-acryloylmorpholine) brushes can be developed for biocompatible systems.

## Supporting information

### Synthesis of S,S′-bis(2-propionic acid)trithiocarbonate

For the synthesis of RAFT agent, S,S′-bis(2-propionic acid)trithiocarbonate (BPAT), CS_2_ (0,059 mol) and KOH (0.082 mol) mixed in 90 mL distilled water and stirred for 5 min. 2-bromo propionic acid (0.031 mol) was added dropwise, stirred for 72 h and extracted with dichloromethane. After extraction, 200 mL of concentrated HCl was added to precipitate product. The yellow solid was extracted with ethyl acetate and dried over anhydrous magnesium sulphate. The solution was filtered and ethyl acetate evaporated under vacuum. The yellow solid was crystallized with toluene, and dried in oven [1].

For the characterization of BPAT, FTIR (Figure S2a), ^1^H-NMR (Figure S2b), ^13^C - NMR (Figure S2c) and TOF-MS (Figure S2d) measurements were performed.

^1^H NMR (300 MHz, CDCI_3_): δ (ppm) 1.6 (d, 3H, -CH3); 4.9 (m, 1H, -CH).

^13^C NMR (300 MHz, CDCI3): δ (ppm) 15.9 (-CH3); 48.6 (-CHS); 170.9 (-C=O); 207.0 (-C=S)

FTIR ν (cm^−1^): approximately 3200 – 2360 (s, -O-H), approximately 2970 (s, aliphatic –C-H), 1693 (s, -C=O), 1100 (s, -C=S).

LC-MS: m/z: [M + H]^+^ calcd for C_7_H_10_O_4_S_3_ 254,29; found 254.98 m/z.

### Synthesis of poly(NAM) via RAFT polymerization

Polymerization of N-acryloylmorpholine were synthesized on Si-surfaces via RAFT polymerization in the presence of AIBN initiator and free BPAT in dioxane at 80 °C. For ^1^H-NMR analysis bulk polymer was precipitated with excess hexane, filtered and dissolved in dioxane. Precipitation-dissolution process was repeated two times, and then polymer was dried in vacuum.

### Determination of optimum irradiation time for covalent attachment of ED and BPAT molecules

Alkene molecules can covalently attach to hydrogen terminated silicon surfaces under UV light. Modification of Si-H wafer with 1,2–epoxy–9-decene were carried for six different irradiation time. Film thickness and water contact angle were measured for each sample and results graphed. With decrease of the water contact angles, the surface hydrophilicity increases and remains constant about at 70° after 4 h and film thickness 1.6 nm (Figure S4a).

To determine optimum reaction time for the BPAT bonding to ED surfaces change of film thickness and water contact angle were followed. It is obvious that as the reaction time increased, the film thickness increased and the water contact angle decreased until the 3rd day. After 3 day of reaction time, film thickness and contact angle did not change significantly. For this reason, the optimum reaction time for the bonding of the BPAT molecules was determined as 3 days (Figure S4b).

Figure S1 Schematic presentation of the synthesis route of the BPAT.

Figure S2 Characterization measurements of BPAT a) FTIR spectra, b) ^1^H-NMR spectra, c) ^13^C-NMR spectra, d) TOF-MS spectra.

Figure S3 ^1^H-NMR spectra of bulk poly(NAM) for 6 h polymerization time.

Figure S4 Change of film thickness and water contact angle depends on reaction times a) Si-ED surfaces, b) Si-BPAT surfaces.

References1

LaiJT
FillaD
SheaR

Functional Polymers from Novel Carboxyl-Terminated Trithiocarbonates as Highly Efficient RAFT Agents
Macromolecules
2002
35
6754
6756
10.1021/ma020362m


## Figures and Tables

**Table 1:** Theoretical and absolute molecular weights of all polymers for all polymerization times.

Polymerization time (h)	Conversion (%)	^1^H-NMR molecular weight (g/mol)	M_n, th_ (g/mol)
2	27	5194	5046
4	59	13194	10726
6	83	16577	14519
8	95	21864	17119

**Table 2:** Atomic concentrations and binding energies XPS for poly(NAM) brushes synthesized for different times.

	O1s		N1s		C1s		S2p		Si2p	
Surface	C=O	C-O	C-N	C=O	C-O/C-S	C-N	C-C/C-H	S-C	S=C	Si
**Time, 2h**										
Energy (eV)	533.1	532.2	400.0	288.0	286.0	286.7	285.0	168.2	167.0	108.0
Conc. (%)	25.2		2.5		58.7			3		10.7
**Time, 4h**										
Energy (eV)	533.1	532.2	400.0	288.0	286.0	286.7	285.0	168.2	167.1	108.0
Conc. (%)	21.0		4.0		66.1			1.5		1.5
**Time, 6h**										
Energy (eV)	533.1	532.2	400.0	288.0	286.0	286.7	285.0	168.2	167.0	108.0
Conc. (%)	18.5		7.2		71.5			1.2		---
**Time,8h**										
Energy (eV)	533.1	532.2	400.0	288.0	286.0	286.7	285.0	168.2	167.0	108.0
Conc. (%)	18.0		7.4		74.6			---		---
