# Supplementary figures and images for: Synthesis and kinetic analysis of poly(N-acryloylmorpholine) brushes via surface initiated RAFT polymerization
Source: Turk J Chem. 2022 Nov 29;47(1):185–95. doi: 10.55730/1300-0527.3528 (PMC10504005; doi:10.55730/1300-0527.3528)

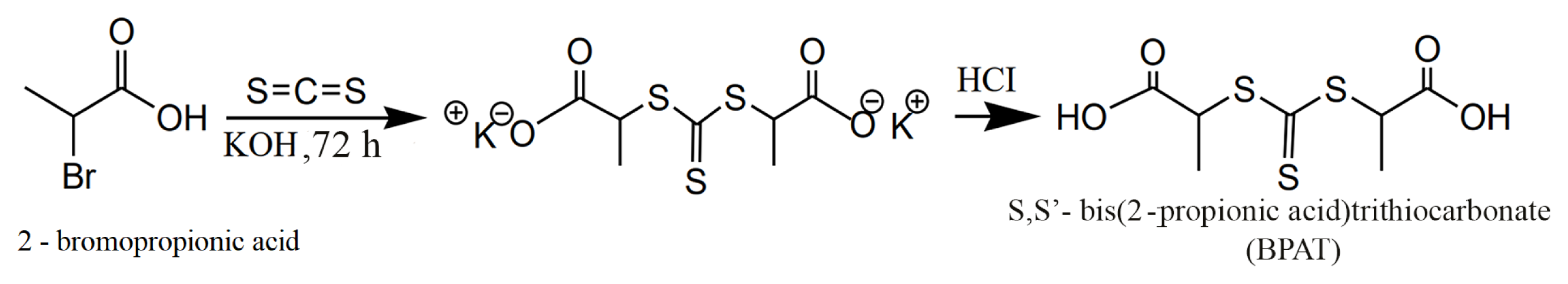

Supplement: Figure S1 — Schematic presentation of the synthesis route of the BPAT. [file turkjchem-47-1-185s1.tif]

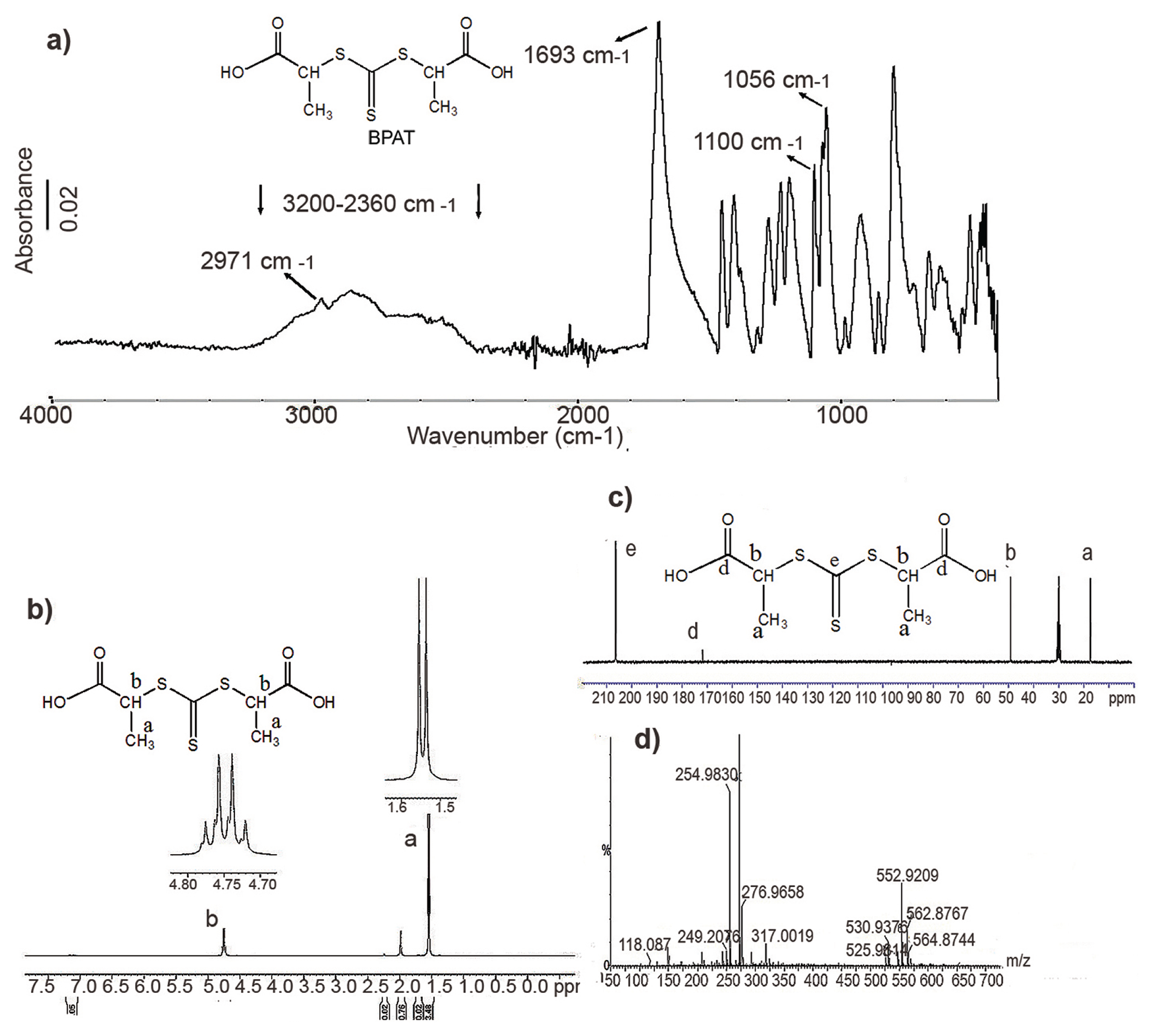

Supplement: Figure S2 — Characterization measurements of BPAT a) FTIR spectra, b) 1H-NMR spectra, c) 13C-NMR spectra, d) TOF-MS spectra. [file turkjchem-47-1-185s2.tif]

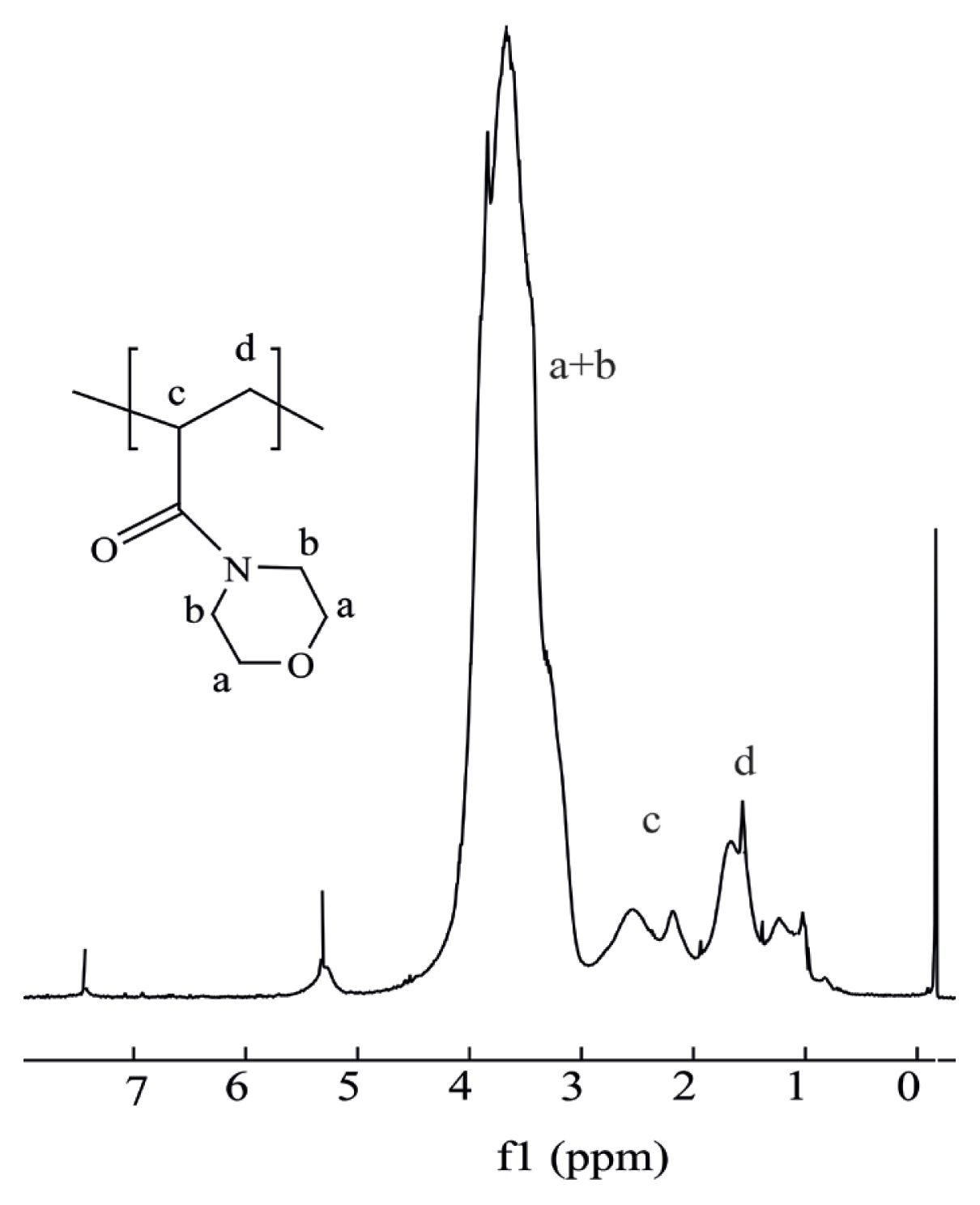

Supplement: Figure S3 — 1H-NMR spectra of bulk poly(NAM) for 6 h polymerization time. [file turkjchem-47-1-185s3.tif]

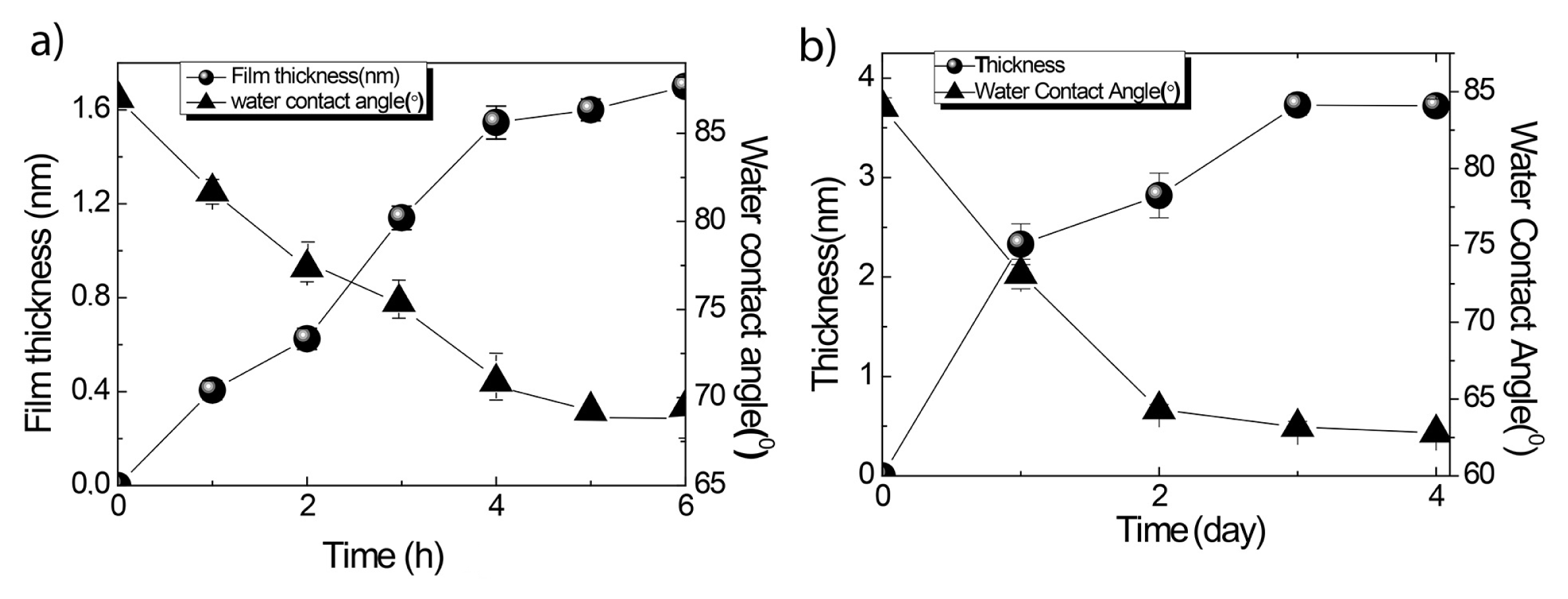

Supplement: Figure S4 — Change of film thickness and water contact angle depends on reaction times a) Si-ED surfaces, b) Si-BPAT surfaces. [file turkjchem-47-1-185s4.tif]
